# Preparation of PMMA Electrospun Fibers Bearing Porphyrin Pendants and Photocatalytic Degradation of Organic Dyes

**DOI:** 10.3390/molecules27238132

**Published:** 2022-11-22

**Authors:** Er-Jun Sun, Xiao-Yan Bai, Yu Chang, Qin Li, Xin-Ru Hui, Yan-Song Li, Yue Wang

**Affiliations:** Key Laboratory of Advanced Green Functional Materials, College of Chemistry, Changchun Normal University, Changchun 130032, China

**Keywords:** metalloporphyrin, polymer, electrospinning, photocatalytic degradation, organic dyes

## Abstract

Porphyrins have a large π–π conjugation force between molecules, and they are easy to aggregate in solution, which affects the photoelectric properties of porphyrins. Connecting porphyrins to polymer links through covalent bonds not only retains the mechanical properties and thermal stability of polymer materials, but also has the photoelectric properties and catalytic properties of porphyrins, which improves the availability of materials. In this study, first, a porphyrin ligand with double bonds in the side chain was designed and the corresponding copper and zinc complexes were synthesized by adjusting the metal ions in the center of the pyrrole ring. Then, the metalloporphyrin complexes were copolymerized with methyl methacrylate (MMA), and two metalloporphyrin/PMMA copolymers were obtained: CPTPPCu/PMMA and CPTPPZn/PMMA. The structure of the compounds was characterized by IR, ^1^H NMR, MS, and UV-Vis spectra. Metalloporphyrin/PMMA copolymers were prepared into electrospun fiber materials by electrospinning. The morphology of the composites was studied by SEM, and the thermal stability and optical properties of electrospun fibers were studied by TGA and FL. The catalytic activity of electrospun fiber materials for the degradation of organic dyes was studied. The results showed that the efficiency of the metalloporphyrin/PMMA copolymer in photocatalytic degradation of methylene blue (MB) was better than that of the PMMA electrospun fiber blended with metalloporphyrin.

## 1. Introduction

Porphyrin is a kind of macromolecular heterocyclic compound which takes the porphin ring as the main body, and the system is highly conjugated [1]. Porphyrins have unique optical properties, which means they play an important role in the field of chemical materials [2]. Porphyrin molecules have a triplet state with appropriate energy. Under light irradiation, they can effectively transfer energy to ground-state oxygen and generate high-yield oxygen. Therefore, they are recognized as very effective photosensitizers [3] and are widely used in medical photodynamic therapy (PDT) [4] and photodynamic inactivation (PDI) of different microorganisms [5]. 

At present, water pollution is a serious environmental problem in society, especially the organic dyes widely used in modern industry, which have the characteristics of large emissions, complex components, not easy to degrade, and do great harm to the environment [6,7,8,9]. Photocatalytic degradation of organic dyes is a relatively effective water treatment technology at present. The commonly used photocatalyst materials include TiO_2_, metal organic framework materials (MOFs), and metalloporphyrin [10,11,12]. However, many photocatalysts represented by TiO_2_ have shortcomings such as relying on ultraviolet light, small particle diameter, and difficult to recover in the experimental process, which limit their wide application [13]. Porphyrin compounds have unique optical properties and are good catalysts for the photooxidation of organic compounds. They can be used as photocatalysts for the degradation of organic dyes in water pollution. However, due to the strong π–π conjugation between molecules, porphyrin monomers are easy to aggregate in solution, which affects their photoelectric properties [14]. Using covalent bonds to connect porphyrins to polymer segments, polymer materials not only retain thermal stability and mechanical properties, but also have special photoelectric properties of porphyrin molecules, improving their applicability [15,16].

**Figure 1:**
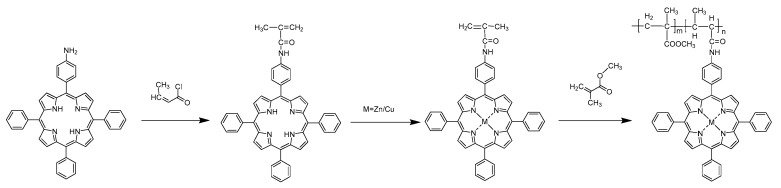
Synthetic route of metalloporphyrin/PMMA copolymer.

In this study, two metalloporphyrin complexes with double bonds in the side chain were designed and synthesized and copolymerized with methyl methacrylate (MMA) to prepare PMMA electrospun fibers bearing porphyrin pendants. The synthesis route is shown in Figure 1. The morphology, optical properties, and thermal stability of the electrospun fibers were studied, and the photocatalytic degradation of organic dyes by porphyrin/PMMA copolymer electrospun fibers under different conditions was also discussed.

## 2. Results and Discussion

### 2.1. Material Characterization

**Figure 2:**
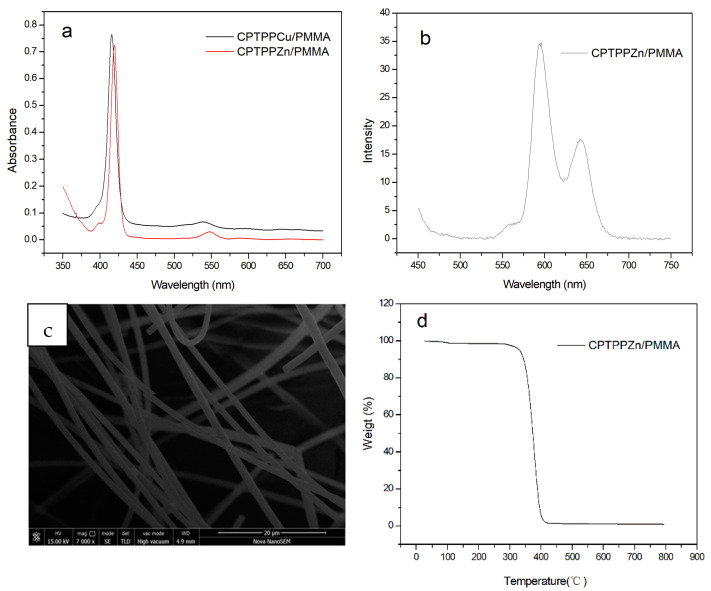
UV-Vis absorption spectrum (**a**), fluorescence emission spectrum (**b**), scanning electron microscope (**c**), and thermogravimetric curve (**d**) of metalloporphyrin/PMMA electrospun fiber.

According to ^1^H NMR, UV-Vis, FT-IR, and MS spectra, the porphyrin monomer CPTPP and metalloporphyrin monomers CPTPPZn and CPTPPCu were the target products. After the formation of metalloporphyrin complexes, the N-H stretching vibration peak and bending vibration peak of CPTPP, about 3310 and 966 cm^−1^, in the infrared spectrum disappeared, the number of Q bands of metalloporphyrins in the UV-Vis absorption spectrum decreased from 4 to 1, and the peak at −2.76 in ^1^H NMR disappeared [17]. The UV-Vis absorption spectra of metalloporphyrin/PMMA electrospun fibers were measured with DMF as a solvent, as shown in Figure 2a. Compared with the metalloporphyrin monomer, the position and number of absorption peaks of the UV-Vis absorption spectrum of the copolymer electrospun fiber have not changed, which proves that the metalloporphyrin is still distributed on each link of PMMA in the form of monomers, and there is no polymerization between the porphyrin monomers. The fluorescence emission spectra of metalloporphyrin/PMMA copolymer electrospun fibers is shown in Figure 2b. The CPTPPZn/PMMA copolymer electrospun fiber has two strong absorption peaks at 595 and 644 nm, which are consistent with the emission peak of the CPTPPZn monomer. Similar to the case of copper porphyrin, no fluorescence emission peak was observed in the CPTPPCu/PMMA electrospun fiber because of the fluorescence quenching [18]. ^1^H NMR was carried out on the copolymer electrospun fiber, but no obvious porphyrin characteristic peak was observed, mainly because the content of metalloporphyrin in the copolymer electrospun fiber material was low, and the instrument could not detect it. The morphology of the metalloporphyrin/PMMA electrospun fiber was observed by SEM, as shown in Figure 2c. The fiber surface was smooth and the diameter distribution was uniform, indicating that the addition of a small amount of metalloporphyrin did not have a great impact on the spinning process, and the electrospun fiber with good morphology could still be obtained.

A small amount of metalloporphyrin/PMMA copolymer electrospun fibers were weighed and tested by thermogravimetric analysis in nitrogen atmosphere. The thermogravimetric curve obtained is shown in Figure 2d. It can be seen that before 250 °C, except for a small amount of solvent and water evaporated by heating, the fiber sample can basically maintain thermal stability without obvious decomposition. However, after 250 °C, the fiber sample began to decompose, and the weight loss was obvious. Near 400 °C, the weight loss of the sample was close to 100%, indicating that the fiber was completely decomposed at high temperature. Theoretically, because the fiber material contains metalloporphyrins, a small amount of metal will remain after the high-temperature decomposition of metalloporphyrins. However, due to the small content of porphyrins in the whole fiber, the residual metal after high temperature is only a trace, which cannot be detected by the instrument. It can be seen from the above data that the electrospun fiber material prepared in this paper has good thermal stability and will not decompose at room temperature or in hot water, which will not affect its use effect [19].

### 2.2. Photocatalytic Degradation under Different Light Sources

**Figure 3:**
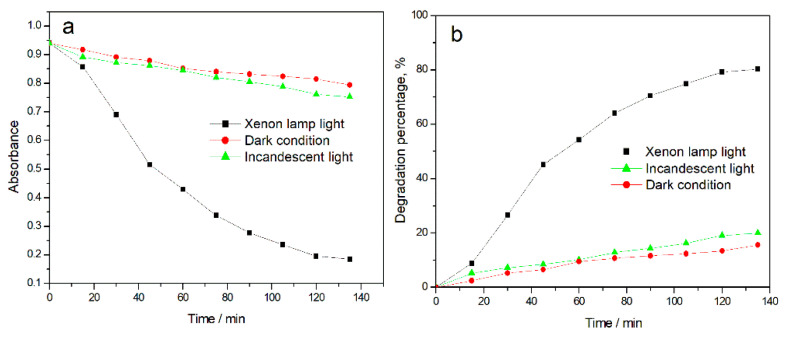
Absorbance curves of MB solution catalyzed by CPTPPCu/PMMA electrospun fiber under different light sources (**a**). Degradation percentage curves of MB solution catalyzed by CPTPPCu/PMMA electrospun fiber under different light sources (**b**).

Under the same conditions, the catalytic degradation activity of metalloporphyrin/PMMA electrospun fibers under different light sources was studied. Here, 100 mL of MB solution and 230 mg of CPTPPCu/PMMA electrospun fiber (equivalent to 1 mg of CPTPPCu) were added to three reactors. After adsorption equilibrium, the three reactors were placed in darkness, incandescent lamp, and xenon lamp conditions for degradation experiments, respectively. The experimental results are shown in Figure 3. Under the xenon lamp condition, CPTPPCu/PMMA electrospun fiber had the best degradation effect on MB solution, and the effect was the most obvious in the first hour, with the degradation percentage reaching 54.29%. After 135 min, the degradation percentages of MB under the xenon lamp, incandescent lamp, and dark conditions were 80.35%, 20.03%, and 15.57%, respectively. Since the xenon lamp light source has a spectrum similar to sunlight, it is a high light power and wide wavelength light source, and the wavelength is continuously distributed, covering ultraviolet, visible, and near-infrared wavelengths. Metalloporphyrins have excellent optical properties, which have strong absorption in visible light (Soret band: 400~450 nm; Q band: 500~700 nm). Therefore, metalloporphyrins show high photocatalytic activity under xenon lamp conditions, and can effectively degrade organic dyes. The incandescent lamp has low energy, metalloporphyrin absorbs less visible light, and its photocatalytic efficiency is low. Under dark conditions, the porphyrin catalyst cannot play a role and has almost no catalytic degradation capacity. Therefore, the xenon lamp was selected as the light source for subsequent photocatalytic degradation experiments.

### 2.3. Photocatalytic Degradation of Different Organic Dyes

**Figure 4:**
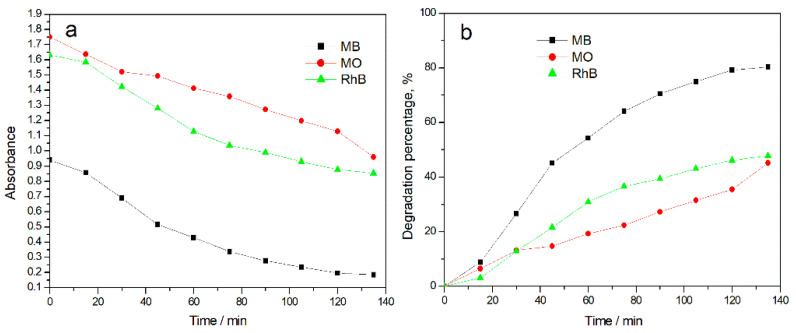
Absorbance curves of CPTPPCu/PMMA electrospun fiber for catalytic degradation of different organic dye solutions (**a**). Degradation percentage curves of different organic dyes catalyzed by CPTPPCu/PMMA electrospun fibers (**b**).

**Figure 5:**
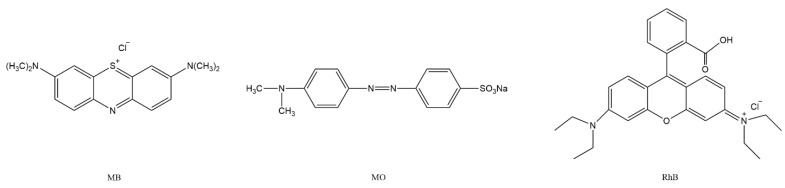
Structural formula of MB, MO, and RhB.

Xenon lamp was used as the experimental light source, CPTPPCu/PMMA electrospun fiber was selected as the photocatalyst, and MB, methyl orange (MO), and Rhodamine B (RhB) were studied as organic dyes for photocatalytic degradation. The degradation percentages were calculated, as shown in Figure 4. The experimental results showed that under the same conditions, the photocatalytic degradation efficiency of the electrospun fiber material CPTPPCu/PMMA for different organic dyes was MB > RhB > MO. At 135 min, the degradation percentages of MB, RhB, and MO were 80.35%, 47.70%, and 45.21%, respectively. Figure 5 shows the chemical structure formula of MB, MO, and RhB. Due to the chromogenic group aniline, =S^+^—in MB is easily damaged by external factors, and MB may demethylated under light irradiation and continue to decompose into smaller organic molecules. During photodegradation, the structure of RhB is destroyed and some unstable colorless organic intermediates are generated, leading to the photodegradation process and discoloration of the solution. However, the acidic azo structure and neutral alkaline quinone structure in MO are relatively stable, which requires more energy to degrade and fade. When it is degraded under ultraviolet and visible light, MO will also undergo demethylation and azo reduction. However, it requires a large amount of energy, which is not easy to achieve. As long as there is a small amount of energy, the structure of MB may change and cause the solution to fade.

### 2.4. Photocatalytic Degradation of Different Catalysts 

To compare the effects of grafting or blending methods on the photocatalytic properties of electrospun fibers, a certain amount of metalloporphyrins were physically blended into PMMA to prepared electrospun fiber materials, which were named CPTPPZn-PMMA and CPTPPCu-PMMA, respectively. In the preparation process, the content of metalloporphyrins blended in PMMA should be the same as that in metalloporphyrin/PMMA copolymer electrospun fibers. In the photocatalytic degradation experiment, 100 mL of MB solution was added to the two reactors, respectively, then 0.23 g of CPTPPCu/PMMA electrospun fibers and an equal amount of CPTPPCu-PMMA electrospun fibers (or 0.37 g of CPTPPZn-PMMA and CPTPPZn-PMMA electrospun fibers) were added, respectively. 

**Figure 6:**
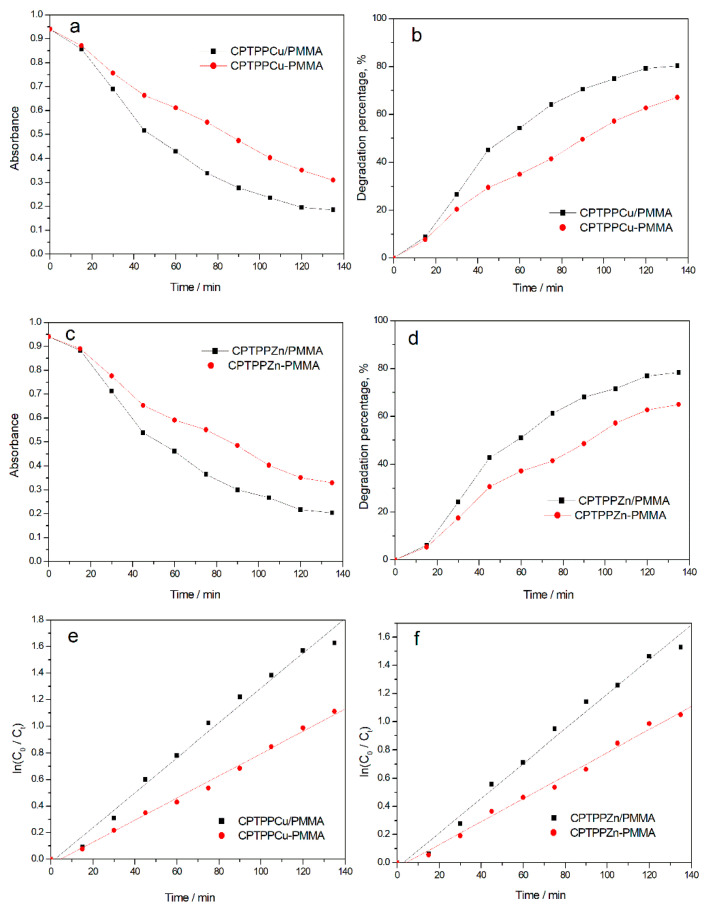
Absorbance curves (**a**,**c**) of MB solution catalyzed by different catalysts. Degradation percentage curves of MB catalyzed by different catalysts (**b**,**d**). Equation of first-order kinetic curve of catalytic degradation of MB with different catalysts (**e**,**f**).

As shown in Figure 6, when the degradation time reached 135 min, the degradation percentage of CPTPPZn/PMMA electrospun fibers reached 78.31%, while CPTPPZn-PMMA electrospun fiber material was only 64.97%. The degradation percentage of CPTPPCu/PMMA electrospun fibers reached 80.35%, while CPTPPCu-PMMA electrospun fiber material was only 67.09%. The catalytic degradation process of all photocatalysts conforms to the standard first-order kinetic equation. The catalytic degradation effect of metalloporphyrin/PMMA copolymer electrospun fibers was significantly better than that of the metalloporphyrin-blended PMMA electrospun fibers. When the electrospun fiber is prepared by blending metalloporphyrin and PMMA, the metalloporphyrin monomer is often poorly dispersed in the polymer matrix. Due to the large conjugated structure of porphyrin, the intermolecular π–π conjugation is strong. Porphyrin monomer is easy to aggregate, and the active site of metalloporphyrin is easily encapsulated by PMMA, so it cannot effectively play a catalytic role. The covalent bond is used to connect the porphyrin monomer to the polymer skeleton to obtain the metalloporphyrin/PMMA copolymer. The metalloporphyrin monomer could be dispersed on the PMMA chain uniformly, which can effectively avoid the aggregation of porphyrins, retain more active sites, and improve the photocatalytic degradation ability of metalloporphyrins [20].

## 3. Materials and Methods

### 3.1. General

UV-Vis spectra were collected on a Cary 300 spectrometer. IR spectra were recorded on a Nicolet iS50 spectrometer using KBr pellets in the region of 4000–500 cm^−1^. ^1^H NMR spectra were recorded on a Varian Unity 400 (400 MHz) NMR spectrometer. Chemical shifts were reported on the *d*-scale relative to tetramethylsilane (TMS). Mass spectra were obtained using a Brucker Autoflex speed TOF/TOF Matrix-Assisted Laser Desorption Ionization Time of Flight Mass Spectrometry. Thermal analysis was recorded on Perkin-Elmer TG-7 apparatus (sample: 3–4 mg, heating rate: 10 °C/min, and nitrogen atmosphere). Fluorescence spectra were recorded with a HITACHI F-7000 spectrofluorometer. SEM figures were collected by the HITACHI SU8100 scanning electron microscope. A single-spindle electrostatic spinning machine was used to prepare electrospun fibers. 

The reagents and solvents used in the experiment were of the commercial analytical pure grade and were used without further purification. The 5-(4-aminophenyl)-10,15,20-triphenylporphyrin was synthesized in the laboratory according to [21].

### 3.2. Synthesis of CPTPP

Here, 200 mg of 5-(4-aminophenyl)-10,15,20-triphenylporphyrin, 30 mL of anhydrous dichloromethane, and 1 mL of triethylamine were added to a three-neck bottle, and nitrogen was injected for 30 min. Then, 1 mL of crotonyl chloride (diluted with 10 mL of anhydrous dichloromethane) was dropped into the bottle dropwise and stirred at room temperature for 10 h. Thin-layer chromatography (TLC) was used to detect the reaction process. After the reaction, the mixture was extracted with saturated sodium bicarbonate solution and distilled water three times, respectively. The dichloromethane solution was concentrated and applied to a silica gel column, and the column was eluted with dichloromethane–petroleum (9:1, *v*/*v*). The first thick purple band was collected and evaporated to obtain CPTPP. UV-Vis (CH_2_Cl_2_) λ: 417 nm (Soret band), 513, 550, 593, 644 nm (Q band); ^1^H NMR (CDCl_3_) *δ*: 8.92–8.88 (m, 8H, pyrrole β-H), 8.30–8.23 (m, 8H, ortho-H phenyl), 8.02–8.00 (d, 2H, para-H phenyl), 7.90 (s, 1H, para-H phenyl), 7.82–7.77 (m, 8H, meta-H phenyl), 6.02 (s, 1H, CH=, Z), 5.65 (s, 1H, CH=, E), 2.25–2.10 (m, 3H, -CH_3_), −2.75 (s, 2H, pyrrole NH) (Figure S1); IR *ν*: 3310, 3045, 3019, 1761, 1599, 1532, 1458, 1352, 966 cm^−1^.

### 3.3. Synthesis of CPTPPZn and CPTPPCu

Here, 200 mg of CPTPP and 30 mL of anhydrous dichloromethane were added to a round-bottom flask equipped with a reflux condenser tube, and the reaction solution was heated to reflux and stirred continuously. Then, 500 mg of zinc acetate (or copper chloride dihydrate) was added, and the reaction time was about 2 h. Reaction solution was washed with distilled water more than three times to remove the unreacted metal salt, dried with anhydrous sodium sulfate, evaporated to dryness, and purified by column chromatography.

The yield of compound CPTPPZn was about 85%. UV-Vis (CH_2_Cl_2_) λ: 415 nm (Soret band), 540 nm (Q band); ^1^H NMR (CDCl_3_) *δ*: 9.02–8.98 (m, 8H, pyrrole β-H), 8.26–8.23 (m, 8H, ortho-H phenyl), 8.01–7.99 (d, 2H, para-H phenyl), 7.90 (s, 1H, para-H phenyl), 7.80–7.78 (m, 8H, meta-H phenyl), 6.01 (s, 1H, CH=, Z), 5.64 (s, 1H, CH=, E), 2.25 (s, 3H, -CH_3_) (Figure S2); IR *ν*: 3051, 3023, 1766, 1598, 1533, 1459, 1355 cm^−1^.

The yield of compound CPTPPCu was about 80%. UV-Vis (CH_2_Cl_2_) λ: 419 nm (Soret band), 549 nm (Q band); IR *ν*: 3048, 3024, 1768, 1595, 1531, 1466, 1349 cm^−1^; MALDI-TOF-MS *m*/*z*: Calcd for C_48_H_33_N_5_OCu 758.20, found 759.20 (Figure S3).

### 3.4. Synthesis of CPTPPZn/PMMA and CPTPPCu/PMMA

According to [22], 50 mg of metalloporphyrin complex, 1700 mg of MMA, and 15 mg of azoisobutyronitrile (AIBN) were dissolved in 15 mL of N, N-dimethylformamide (DMF). The solution was placed in a two-neck bottle, degassed three times, and polymerized for 24 h under the condition of a sand bath of 90 °C. The viscous solution was poured into distilled water and the precipitated polymer was separated by filtration. The polymer was placed in a Soxhlet extractor and extracted with chloroform for 48 h until the washed solution was colorless, to allow full removal of the residual porphyrin monomer. The polymer was then dried under vacuum, and two metalloporphyrin/PMMA copolymers were obtained. 

### 3.5. Preparation of Metalloporphyrin/PMMA Electrospun Fiber Materials

According to [23], the metalloporphyrin/PMMA copolymer solution with a mass concentration of 30% was prepared with DMF as a solvent, and stirred for 6 h to produce a homogeneous, stable, and viscous spinning solution. The configured spinning solution was injected into a 10 mL syringe. A positive voltage (18 kV) was applied to the polymer solution and the distance between the syringe tip and the collector surface was ca. 20 cm. The flow rate of the polymer solution was kept at 1.0 mL/h. After spinning, the tin foil was removed and it was put into the oven at 100 °C to evaporate the solvent, and then CPTPPCu/PMMA and CPTPPZn/PMMA electrospun fiber materials were obtained. By measuring the absorbance of a certain concentration of electrospun fiber solution, the content of metalloporphyrins in metalloporphyrins/PMMA electrospun fiber materials can be calculated according to the concentration absorbance standard curve, in which the content of CPTPPZn was 0.27%, and the content of CPTPPCu was 0.43%.

### 3.6. Photocatalytic Degradation Experiment

The photodegradation experiment was carried out using a Model HSX-F300 photocatalytic reactor with a 300 W Xenon lamp simulating sunlight. The average light intensity on the sample surface was about 700 mW·cm^−2^. The temperature inside the reactor was maintained at 25 °C with a circular water running via an interlayer. Then, 100 mL of MB, MO, and RhB aqueous solutions with the concentration of 2.5 × 10^−5^ g/L were studied as the degraded samples. Metalloporphyrin/PMMA electrospun fibers were used as a photocatalyst and added to the reactors. The resulting mixture were stirred for 1 h under dark condition to disperse evenly before irradiation with a 300 W Xenon lamp or 40 W incandescent light bulb. The related reaction progress was monitored by UV-Vis absorption spectra every 15 min. The degradation percentage was calculated according to the following formula [24]: degradation percentage = (A_0_ − A_t_)/A_0_ × 100%, where A_t_ represents the absorbance at 664 nm for MB (463 nm for MO and 554 nm for RhB) when the reaction time is t, and A_0_ represents the absorbance at 664 nm for MB solution at the initial time.

## 4. Conclusions

In this study, metalloporphyrins were grafted onto PMMA side chains through copolymerization, and two kinds of metalloporphyrins/PMMA electrospun fiber materials were prepared by electrospinning technology. SEM and TG studies showed that the synthesized electrospun fiber materials had good fiber morphology and good thermal stability. The photocatalytic degradation of organic dyes by electrospun fiber materials was studied in detail, and the effects of different light sources on the catalytic degradation of organic dyes by electrospun fiber materials were explored. The results showed that the degradation effect was xenon lamp > incandescent lamp > dark condition. The photocatalytic degradation effect of CPTPPCu/PMMA photocatalysts on different organic dyes was investigated. The results showed that the order of photocatalytic degradation activity of photocatalysts on different dyes was MB > RhB > MO. The photocatalytic degradation of organic dyes of PMMA electrospun fibers blended with metalloporphyrin and metalloporphyrin/PMMA copolymer electrospun fibers was compared. The results showed that the efficiency of the metalloporphyrin/PMMA copolymer in photocatalytic degradation of MB was better than that of the PMMA electrospun fiber blended with metalloporphyrin.

## Data Availability

Data are contained within the article and Supplementary Materials.

## Supplementary Materials

The following supporting information can be downloaded at: https://www.mdpi.com/article/10.3390/molecules27238132/s1, Figure S1: ^1^H-NMR spectra for CPTPP. Figure S2: ^1^H-NMR spectra for CPTPPZn. Figure S3: MS spectra for CPTPPCu.

## Sample Availability

Samples of all compounds are available from the authors.
