# Supplementary material for: Preparation of PMMA Electrospun Fibers Bearing Porphyrin Pendants and Photocatalytic Degradation of Organic Dyes
Source: Molecules. 2022 Nov 22;27(23):8132. doi: 10.3390/molecules27238132 (PMC9738649; doi:10.3390/molecules27238132)
Supplement: Supplementary file 1 [file molecules-27-08132-s001.zip › molecules-1950395-supplementary.pdf]

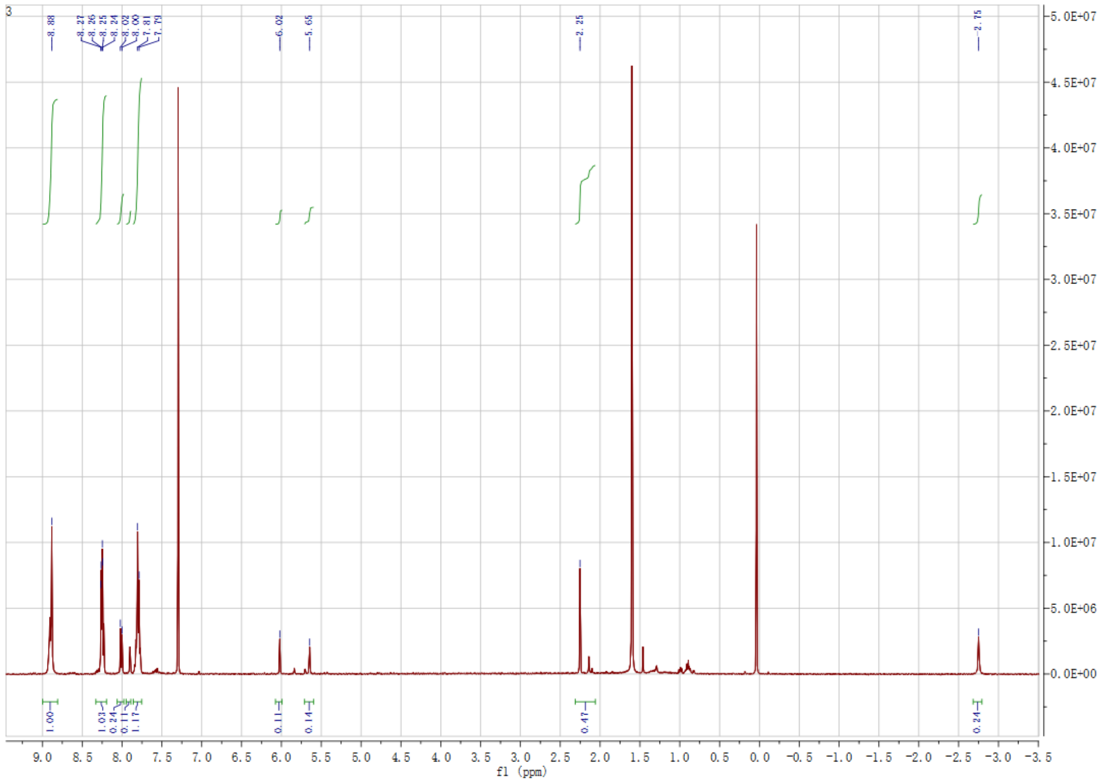

Figure S1. ^{1}H-NMR Spectra for CPTPP.

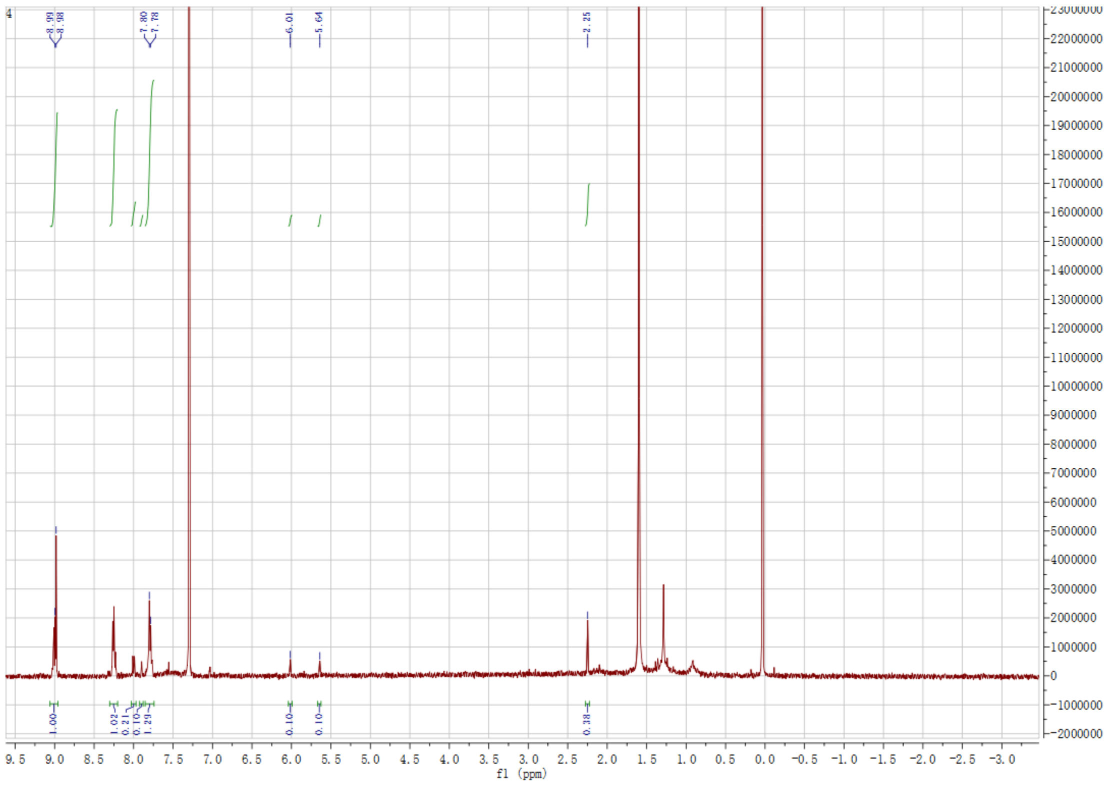

Figure S2. ^{1}H-NMR Spectra for CPTPPZn.

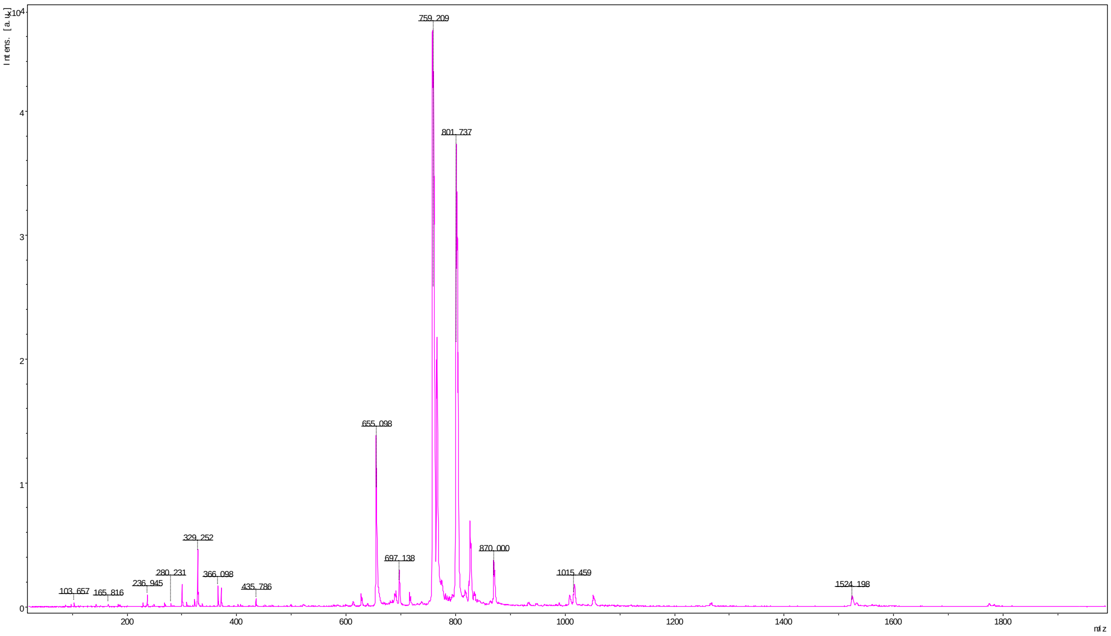

Figure S3. MS Spectra for CPTPPCu.
